# A Phosphorous-Containing Bio-Based Furfurylamine Type Benzoxazine and Its Application in Bisphenol-A Type Benzoxazine Resins: Preparation, Thermal Properties and Flammability

**DOI:** 10.3390/polym14081597

**Published:** 2022-04-14

**Authors:** Chunxia Zhao, Zhangmei Sun, Jixuan Wei, Yuntao Li, Dong Xiang, Yuanpeng Wu, Yusheng Que

**Affiliations:** 1 School of New Energy and Materials, Southwest Petroleum University, Chengdu 610500, China; aa2515954054@163.com (Z.S.); wjx15076120896@163.com (J.W.); d.xiang@swpu.edu.cn (D.X.); wuyp362@126.com (Y.W.); m15351372549@163.com (Y.Q.); 2 State Key Laboratory Oil and Gas Reservoir Geology and Exploitation, School of New Energy and Materials, Southwest Petroleum University, Chengdu 610500, China; 3 The Center of Functional Materials for Working Fluids of Oil and Gas Field, School of New Energy and Materials, Southwest Petroleum University, Chengdu 610500, China

**Keywords:** polybenzoxazine (PBa), cone calorimeter (CONE), limited oxygen index (LOI)

## Abstract

Polybenzoxazine (PBa) composites based on phosphorous-containing bio-based furfurylamine type benzoxazines (D-fu) and bisphenol-A type benzoxazines (Ba) were developed for flame retardation. The structure of D-fu was analyzed by Fourier transform infrared (FTIR) spectroscopy and ^1^H-NMR spectroscopy. The curing temperature of Ba/D-fu mixtures was systematically studied by differential scanning calorimetry (DSC). Thermogravimetric analysis (TGA) demonstrated the excellent char formation ability of the PBa composites with the addition of phosphorous-containing D-fu. The flame retardancy of the PBa composite materials was tested by the limited oxygen index (LOI), vertical burning test (UL-94) and cone calorimeter (CONE). The LOI and UL-94 level of PBa/PD-fu-5% reached 34 and V0 rate, respectively. Notably, the incorporation of 5% D-fu into PBa led to a decrease of 21.9% at the peak of the heat-release rate and a mass-loss reduction of 8.0%. Moreover, the fire performance index increased, which demonstrated that the introduction of D-fu can diminish fire occurrence. The role of D-fu in the condensed and gas phases for the fire-resistant mechanism of the PBa matrix was supported by SEM-EDS and TGA/infrared spectrometry (TG-FTIR), respectively. Dynamic mechanical analysis (DMA) revealed that the Tg of PBa flame-retardant composites was around 230 °C. Therefore, PBa composites are promising fire-retardant polymers that can be applied as high-performance functional materials.

## 1. Introduction

Polybenzoxazine(PBa), as a novel high-performance phenolic resin, is prepared by formaldehyde compound, primary amines and phenols [1,2,3,4]. PBa is widely used in military, aeronautics and electronics [5,6,7,8,9,10] due to its flexibility in molecular design, catalyst-free polymerization, good mechanical properties and high glass-transition temperature [11,12,13,14,15,16]. However, the limited oxygen index (LOI) of bisphenol A-based benzoxazine (Ba) is only 21, which indicates that it is easily flammable and, therefore, requires modifiying to enlarge its range of applications [17]. In the past few years, there have been two main strategies for the modification of benzoxazines to reduce their combustibility. Chemically-intrinsic flame retardance has been achieved by introducing flame-retardant elements into the chemical structure of Ba to enhance the flame-retardance performance of PBa resins. Yan et al. prepared bio-based Ba using furfurylamine (FA) as an amine source and phloretic acid (PHA), p-coumaric acid (pCOA) and ferulic acid (FEA) as phenol sources [18]. The as-obtained bio-based PBa resins exhibited high residual char at 800 °C, low heat release capacity and total heat release values. Meanwhile, the bio-originated compounds, i.e., sesamol, furfurylamine and benzaldehyde, were employed to fabricate a new kind of PBa, showed a high char formation, high degradation temperature and low heat release capacity [19]. Mei et al. prepared a novel Ba monomer by incorporating both acetylene groups and silicon elements within the chemical structure of Ba. The corresponding experimental results demonstrated good flame retardant properties with 80% char yields at 1000 °C in N_2_ and 21% in air atmosphere, respectively [20]. In recent years, the development and application of halogen-free flame-retardant additives is an important topic in various fields for meeting the requirements of sustainable development [21,22,23]. PBa composites based on hexa-(4-amino-phenoxy)-cyclotriphosphazene (CPA) showed improved ablative performance and high char yield [24]. For instance, Bz-MMT was modified via an ion-exchange reaction between Na-montmorillonite (Na-MMT) and benzoxazine. Following this, PBZ/Bz-MMT was prepared by heating the mixture of BZ monomer and MMT, improving the char yield, tensile strength and impact strength of PBZ/Bz-MMT nanocomposites [25]. Spontonet et al. successfully prepared fire retardant PBa resins via the crosslinking reaction of DOPO-containing Ba monomers and bisphenol A-based Ba monomers. The LOI values of the composites were increased by 8.2 with an increasing phosphorus content of 7.8 [26]. According to Selvia’s research, cyclophosphazene nanotube (PZT) incorporated poly(benzoxazine-co-ε-caprolactam) (P(BZ-co-CPL)) nanocomposites were prepared to improve their flame retardant properties. The LOI value was raised to 31.4 with 1.5 wt.% PZT in the composite matrix [27]. PG-FA, PG-A, and di-benzoxazines based on pyrogallol furfurylamine and pyrogallol aniline showed an obvious enhancement in the char layer formation at 800 °C and a reduction in the ring-opening polymerization (ROP) temperature [28]. Moreover, intumescent flame retardants (IFR), i.e., phosphorus–nitrogen (P–N) containing flame retardants, were extensively used in the above-reported polymer composites on account of their high efficiency, environment-friendly nature and convenience. In addition, IFR-containing composites can produce a stable carbon-based protective layer, which holds back flammable gases during pyrolysis and isolates the heat from the unburned matrix. Furthermore, phosphorus-containing radicals tend to react with H· and HO·radicals in the gas phase to discontinue a series of reflection. Specifically, phosphorus–nitrogen flame retardant can generate nonflammable NH_3_ gases during the thermal degradation process of polymers, affecting the flammability of a material [29,30,31]. The development and application of renewable resources has been an important topic in many fields due to the requirements of environment protection and petroleum resource depletion. Hence, employing renewable raw materials to fabricate environment-friendly flame retardant reagents has become an important research field. Recently, according to the literature, bio-based polymers, such as phosphated cellulose (PCF), chitosa, lignosulfonate and starch, were used to improve the fire resistance performance of polymers.

Herein, the important aims of this work are to study the effect of D-fu on the flame retardant properties and to study the flame-retardant mechanism of PBa. D-fu is a type of biological derived filler, synthesized by using 10(2,5-Dihydroxypheny)-10h-9-oxa-10-phospha-phenanthrene-10-oxide (DOPO-HQ), paraformaldehyde and furfurylamine. The influence of the addition of D-fu on the performance of composites is investigated using DSC, TGA, CONE, LOI, UL-94 and DMA. In addition, a possible flame retardant mechanism is proposed based on charred layer analysis and TG-FTIR results.

## 2. Experimental Section

### 2.1. Materials

Bisphenol-A type benzoxazine monomer (Ba) was provided by Sichuan Tiance Jucai Technology Co. Ltd., (Chengdu, China). 10(2,5-Dihydroxypheny)-10h-9-oxa-10-phospha-phenanthrene-10-oxide (DOPO-HQ) was purchased from Shanghai Xushuo Biotechnology Co. Ltd., (Shanghai, China). Furfurylamine was obtained from Chengdu Best Reagent Co. Ltd., (Chengdu, China). Paraformaldehyde (95%), toluene, ethanol and acetone were obtained from Chengdu Kelong Chemical Reagent Factory (Chengdu, China). The above chemicals were all used as received without further purification.

### 2.2. Preparation of Benzoxazine Monomer of Furfurylamine (D-fu)

**Figure 1:**
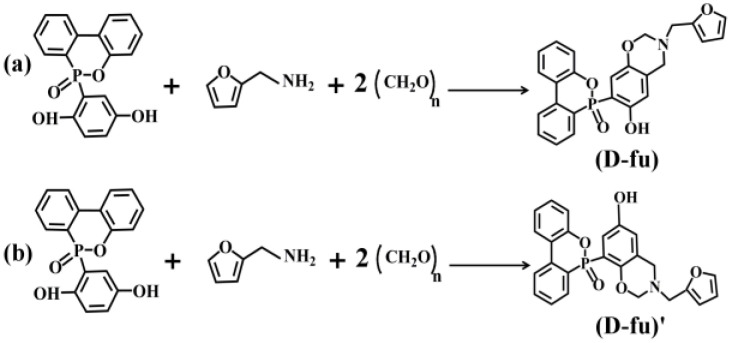
The possible chemical-reaction pathways of furfurylamine type benzoxazine monomer of (**a**) and (**b**).

First, DOPO-HQ (0.01 mol, 3.2027 g), paraformaldehyde (0.02 mol, 0.6006 g) and furfurylamine (0.01 mol, 0.9712 g) were added to 100 mL toluene and 50 mL ethanol in a three-neck bottle (250 mL). The synthesis of D-fu was then carried out at 120 °C for 36 h. The initial products were concentrated with a reduced pressure to remove the organic solvents after the reaction system had been cooled to ambient temperature. The D-fu benzoxazine monomer was dried under ambient pressure. The final benzoxazine of furfurylamine exhibited a light-yellow color. The possible chemical-reaction pathways of D-fu type benzoxazine monomers are shown in Figure 1.

### 2.3. Preparation of PBa/PD-fu Composites

**Figure 2:**
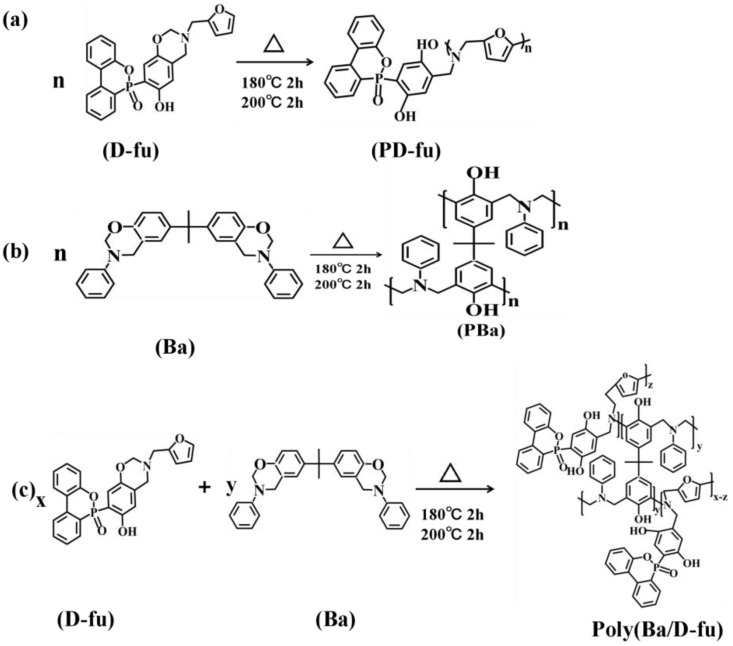
The ideal chemical structure and curing schematic diagram of (**a**) D-fu, (**b**) Ba and (**c**) the crosslinking reaction of D-fu and Ba.

First, 0.87 g of D-fu and 87 g of Ba were fluxed with 35 mL acetone with a single neck bottle (250 mL) at room temperature. Acetone was then removed using a rotary evaporator. Finally, the hybrids were dumped into a glass mold and pre-cured at 180 °C/2 h and further reacted at 200 °C for 2 h. The final material was marked as PBa/PD-fu-1%. Neat PBa, PBa/PD-fu-3% and PBa/PD-fu-5% were manufactured using a similar operation process. The ideal crosslinking reaction schematic diagram of D-fu and Ba are shown in Figure 2. The ring-opening crosslinking reaction of D-fu isomers undergoes a similar route.

### 2.4. Characterization

The chemical structure of D-fu was analyzed by Fourier transform infrared (FTIR) spectroscopy using a Nicolet 6700 spectrometer (Thermo Electric Corporation, Waltham, MA, USA) with the scope of 4000–500 cm^−1^. Moreover, ^1^H-NMR spectroscopy was recorded by an NMR spectrometer (Bruker, 400 MHz) using dimethylsulfoxide (DMSO) as the solvent and tetramethylsilane (TMS) as the inner standard. The morphology was observed by a scanning electron microscope (SEM, ZEISS EV0 MA15). Thermal stability was investigated using a thermogravimetry analyzer (DSC823 TGA/SDTA85^e^) under N_2_ and air atmosphere, respectively. The temperature rate and range were 20 °C min^−1^ and 40 °C to 800 °C, respectively. Differential scanning calorimetry (DSC) measurements were recorded using the non-isothermal DSC Q20 (TA Instruments) at a scanning rate of 10 °C·min^−1^ from 40 °C to 300 °C. The fire resistance property was analyzed through cone calorimetry (CONE, ASTME1354/ISO 5660). The dimension of the sample was 100 m × 100 m × 3.2 m; the sample was encompassed in an aluminum foil and tested at a heat flow of 35 kW·m^−2^. Moreover, dynamic mechanical analysis (DMA) was performed using a Q800 analyzer (TA Instruments) in the temperature range of 40–300 °C at a rate of 5 °C/min with a frequency of 1 Hz in air. The sample size was 40 m × 10 m × 3.2 m. Thermogravimetric analysis coupled with Fourier transform infrared (TG-FTIR) was carried out, and the sample was tested with a Perkin-Elmer STA 6000 in nitrogen and air atmospheres with a heating rate of 10 °C/min from 40 °C to 800 °C. The limiting oxygen index (LOI) was measured using an HC-2C Oxygen Index Flammability Gauge (Jiangning, China) according to the ASTM D2863–97 standard, the sizes of all samples were 130 mm × 3.5 mm × 3.2 mm. The vertical burning test (UL-94) was carried out using a GZF-5 instrument (Wuhan, China) according to the GB/T 8333–2008 standard. For the test, the sizes of all samples were 125 mm × 12.7 mm × 3.2 mm. The structure of char residue gained from the CONE test was analyzed using Raman spectroscopy (ID Raman micro IM-52, OceanOptics) (Shanghai, China) equipped with a laser wavelength of 785 nm.

## 3. Results and Discussion

### 3.1. Characterization of D-fu

**Figure 3:**
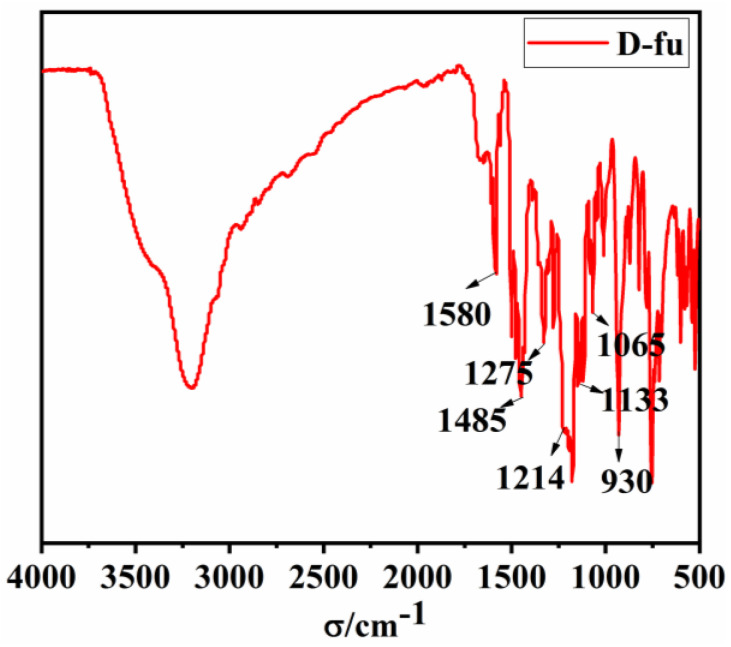
FITR spectrum of D-fu.

**Figure 4:**
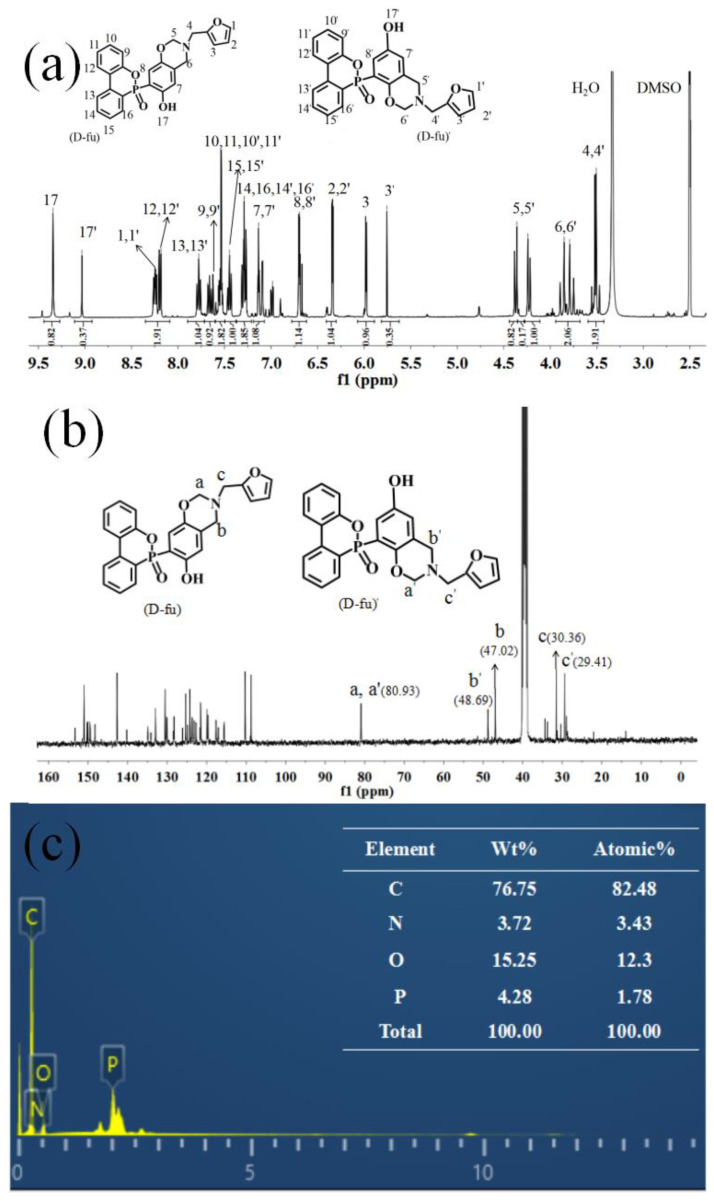
(**a**) ^1^H−NMR spectra, (**b**) ^13^C−NMR spectra and (**c**) EDS spectra of the D-fu.

The chemical structure of D-fu was confirmed by FT–IR, ^1^H–NMR, ^13^C–NMR and EDS analyses, as shown in Figure 3 and Figure 4, respectively. In the FTIR spectrum, the distinctive peaks at 1485 and 1580 cm^−1^ are attributed to the aromatic C=C double bonds. Meanwhile, the peak at 1275 cm^−1^ is due to the CH_2_ wagging. The characteristic peaks at 1065 and 1214 cm^−1^ are associated with the C–O–C asymmetric and symmetric stretching absorptions, respectively [29,30]. The particular peak of 1133 cm^−1^ results from the vibrations of the asymmetric stretching of the chemical bonds in C–N–C groups of the oxazine ring. The peak at 930 cm^−1^ belongs to the characteristic out-of-plane absorption of a benzene group with an attached oxazine ring. The above results support the successful formation of oxazine rings.

The characteristic peaks of the oxazine ring in the benzoxazine monomer exist in the range of 4.35 ppm (O−CH_2_−N) [31] to 3.85 ppm (Ar−CH_2_−N), which can be seen in Figure 4a. The signals at 6.69–7.78 ppm belong to the hydrion of –OH on the aromatic ring. The methylene protons (–CH_2_) of furfurylamine, connecting with the oxazine ring, can be identified from the peak of 3.51 ppm [32]. The furan ring displays resonances at 8.25 ppm, 6.343 ppm and 5.98 ppm. More importantly, the integral area of H_17_ and H_17′_ is at a ratio of 2:1, which means the existence of isomers for furfurylamine-type benzoxazine monomers. Thus, the assignments of ^1^H–NMR signals are consistent with the existence of two D-fu isomers. The D-fu structure was then further confirmed by a ^13^C–NMR study. Figure 4b shows the ^13^C–NMR spectrum for D-fu. The peaks observed at 48.69, 47.02 and 80.93 ppm are assigned to Ar–CH_2_–N (b, b’) and –O–CH_2_–N(a, a’) carbons, which confirm the oxazine ring formation. The peaks appearing at 30.36 and 29.41 ppm correspond to the methylene protons (–CH_2_) of furfurylamine that connect with the oxazine ring (c, c’). Furthermore, as shown in Figure 4c, an EDS test was performed to detect the existence of P atoms with a content about 4.28% in D-fu, which is in agreement with the theoretical calculation for P atoms based on the chemical structure of the D-fu molecule. The above analysis and discussion confirm the successful synthesis of P-containing D-fu.

### 3.2. Curing Behavior of Ba and Ba/D-fu Mixture

**Figure 5:**
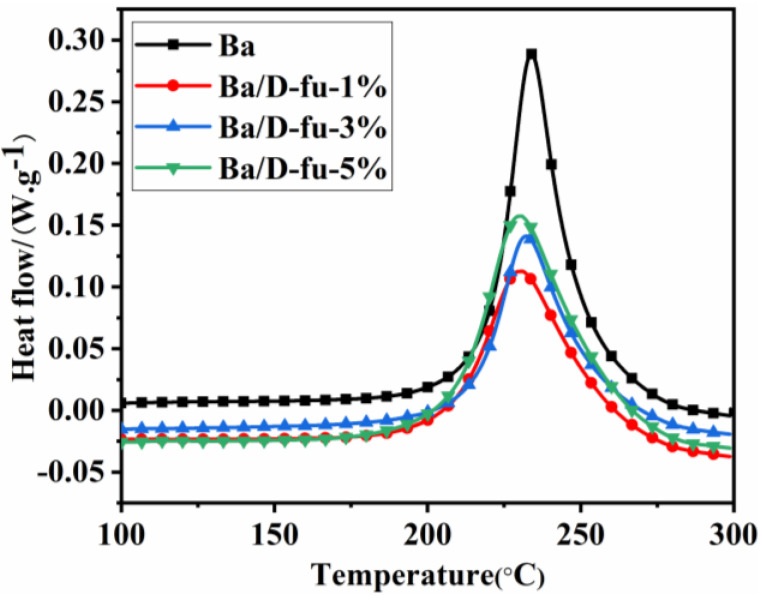
DSC curves for the Ba and Ba/D-fu systems at a scanning pace of 10 °C/min.

The thermal polymerization behaviors of the Ba and Ba/D-fu systems were investigated and corresponding results are shown in Figure 5. The characteristic curing parameters, such as the initial polymerization temperature (T_i_), maximum polymerization temperature (T_p_) and final polymerization temperature (T_f_) are summarized and listed in Table 1. Curves in Figure 5 show one exothermic peak of the ring-opening thermal reaction of the Ba and Ba/D-fu mixture. Owing to the addition of D-fu, both the T_i_ and T_f_ of Ba/D-fu are slightly decreased. Compared with Ba, both the T_i_ and T_f_ of Ba with 5 wt.% D-fu are reduced by 6 °C and 5 °C, respectively. However, the T_p_ of Ba/D-fu-5% remains almost the same. In general, the empty electron orbits of the P atoms around nuclei can receive the duplets of O atoms on the benzene ring, leading to the formation of coordination linkage in the presence of D-fu. The hydrogens of a phenolic hydroxyl group can easily form active H on account of the electron-withdrawing of the P=O bonds, and −OH can accelerate the curing of PBa [33,34]. Thus, the induction time in the curing process of Ba was shortened and the reaction rate was effectively accelerated. Finally, the curing process was maintained at 180 °C/2 h, followed by 200 °C/2 h, to achieve the complete curing process.

### 3.3. Thermal Stability

**Figure 6:**
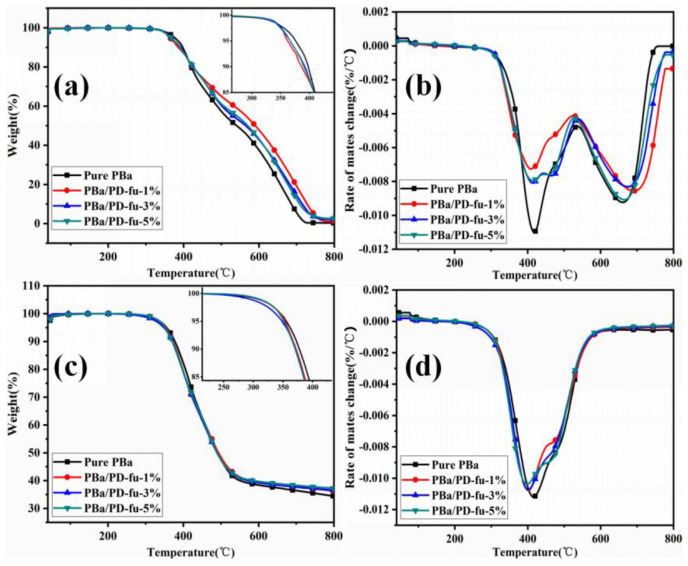
(**a**,**c**) TGA and (**b**,**d**) DTG curves for PBa and PBa/PD-fu composites in air and N_2_ atmospheres.

TGA was used to analyze the thermal decomposition behaviors of PBa/PD-fu composites. The TG and DTG curves in air and N_2_ environments are given in Figure 6. The onset decomposition temperature (T_onset_) refers to the temperature with a mass loss of 5 wt.% [35]. The temperature of the maximum weight rate (T_max_) and amount of char yields are listed in Figure 6a–d. Meanwhile, the detailed data are summarized in Table 2. From Figure 6 and Table 2, it can be inferred that the incorporation of D-fu lowers both the T_onset_ and T_max_ of decomposition. The T_onset_ decreases from 376 °C for the original matrix to 362 °C for PBa/PD-fu-1% in air atmosphere, and from 408 °C to 389 °C in N_2_, respectively. Meanwhile, compared with neat PBa, both the T_onset_ and T_max_ of PBa/PD-fu-5% decrease to 366 °C and 378 °C in air, and 351 °C and 380 °C in N_2_, respectively. The charred residues of composites after the addition of D-fu showed higher increments, especially at high temperatures. It is observed that the char residues of the neat PBa and PBa/PD-fu-1% at 800 °C were 0.34% and 1.26% in air, respectively. The char residue content of PBa/PD-fu-5% at 800 °C is slightly higher than that of PBa/PD-fu-1% in air. It is noted that D-fu leads to a more efficient enhancement in the quantity of residue at 700 and 800 °C, notably in air. This can be attributed to the fact that –N=C=O groups of PBa are generated by the reaction of O_2_ during the degradation process of Mannich-based linkages, which further forms stable crosslinking structures and promotes the formation of a higher amount of char [36,37]. It is evident that the addition of D-fu enhances thermal stability.

The composites and pure PBa exhibit a two-stage decomposition pattern in the air. The thermal degradation of all polymers primarily occurs in the temperature range of 380 to 750 °C. The first stage is between 380 and 450 °C, corresponding to a strong DTG peak around 415 °C on account of the decomposition of phenols and amines in the PBa matrix while burning in the N_2_ atmosphere. Meanwhile, the –CH_2_–N (–R)– group is the thermally “fragile link” of the original material, which leads to middle decomposition of neat material. The weak O=P and P–O bonds of D-fu can promote the rapid pyrolysis of PBa, leading to a lower T_onset_ of PBa/PD-fu composites [38]. During the heating process, the P-containing groups decompose to react with PBa, accelerating the decomposition process of polymers to apparently increase the yield of char residues [39].

Furthermore, the phosphorous content speeds up the formation of stable charred residue, resisting the heat and substance transfer, thereby protecting char from further oxidation and degradation. The second stage can be observed in the temperature range of 600 to 780 °C in the air. This phenomenon may be caused by the phenolic hydroxyl groups in polymer, which are apt to form oxidative crosslinkings in air, and lead to further thermal decomposition, taking the shape of a firm char layer. The char layers on the surface of PBa can deter the propagation of O_2_ and inhibit the outflow of thermal decomposition products. The results of TGA analysis indicate that the addition of D-fu enhances the thermal stability of PBa and generates stable char structures.

### 3.4. Flammability of PBa and PBa/D-fu Composites

The influence of D-fu on flame retardance of PBa was evaluated by UL-94 vertical tests and the LOI. The results of the LOI and UL-94 tests are summarized in Table 3. The LOI value of pure PBa was 21. The flame retardance of the PBa resins is prominently promoted by the addition of D-fu. The self-extinguishing of PBa polymers with loadings of 3 wt.% and 5 wt.% can be observed and the burning time is markedly reduced. In particular, the LOI value of PBa/PD-fu-5% is leveled at 34. Moreover, the UL-94 level increases to the V-0 grade. These phenomena reveal that the incorporation of D-fu into polymers can enhance their fire-retardant performance.

**Figure 7:**
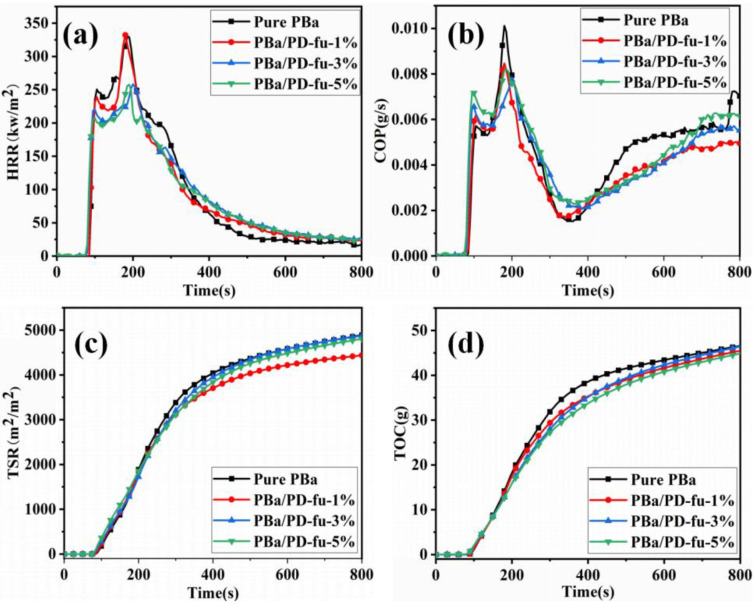
(**a**) HRR, (**b**) COP, (**c**) TSR and (**d**) TOC with respect to time for PBa and PBa/PCF composites based on the CONE calorimetry.

Moreover, flammability characteristics and the potential fireproof safety of polymers under well-ventilated conditions were determined using the cone calorimeter (CONE) with the aim of studying the properties of PBa polymers during combustion. Figure 7 shows the parameters of assessing flame retardancy, including the heat release rate (HRR), carbon monoxide production (COP), total smoke release (TSR) and total oxygen consumption (TOC). The corresponding time to ignition (TTI), peak of HRR (PHRR), mass-loss rate, and fire-growth rate index (FPI) are listed in Table 3.

TTI shows the time the sample resting on the material takes to ignite below the thermal current of the CONE test. It should be noted that the TTI of PBa/PD-fu composites is shorter than the neat PBa (Table 3). Specifically, the TTI value of neat PBa is 83 s, which is much longer than the value of 75 s for PBa/PD-fu-5%. This is mainly caused by the presence of less-stable P-O and P=O groups, which are produced from D-fu, leading to the shortening of the TTI. The shorter TTI can arrest or avert continuous combustion, strengthen the carbon structure, and reduce the decomposition of PBa [40,41,42]. PBa/PD-fu-5% displays a mass loss of 71.7% (Table 3), which is lower than 77.9% for the neat PBa. The fire performance index (FPI) is the ratio of the TTI to PHRR, which is used to estimate the dedication of polymers to the fire [43]. The index of FPI of composites increases, indicating the diminished occurrence of fire with the addition of D-fu into composite matrix.

**Figure 8:**
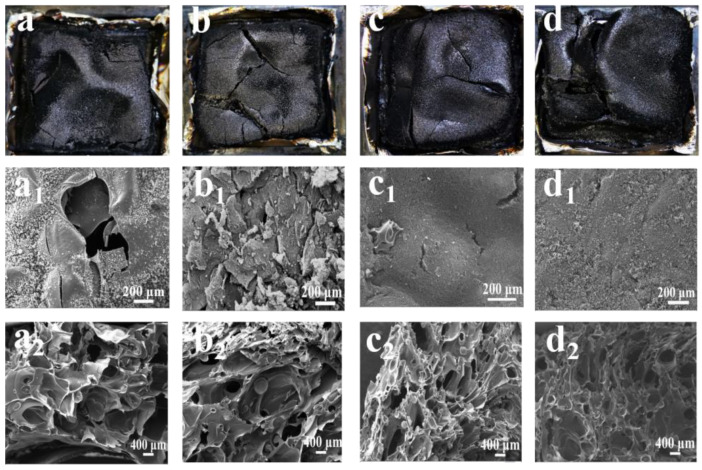
Digital photos of the charred layers (**a**–**d**) and SEM images of the carbon residues outside regions (**a_1_**–**d_1_**) and internal structures (**a_2_**−**d_2_**) of (**a**) PBa, (**b**) PBa/PD-fu-1%, (**c**) PBa/PD-fu-3% and (**d**) PBa/PD-fu-5%.

It has been reported that the HRR and PHRR are two most decisive parameters to study the fire safety of flame-retardant polymers. The HRR and PHRR provide the fuel-feeding rate during burning and the rate of fire spread. Normally, a lower PHRR means a slower flame spread and less fire risk. Figure 7a and Table 3 show that the introduction of D-fu in PBa reduces the HRR values significantly. There are two peaks for composites: the first PHRR (PHRR_1_) of PBa/PD-fu-5% is about 205.9 kW/m^2^, whereas the second PHRR (PHRR_2_) of PBa/PD-fu-5% is 257.8 kW/m^2^. Both are lower than that of pure PBa. It is clear that the significant reduction in PHRR is due to the introduction of D-fu forming cross-linking char of composites. The production of CO in fire is mainly because of incomplete burning. Figure 7b shows that the COP of PD-fu-5% is obviously low during combustion, which implies that the adjunction of D-fu can reduce the production of CO and toxicity of materials during burning. As shown in Figure 8c, the TSR values of PBa composites are also reduced compared with those of neat PBa; this implies that the introduction of D-fu can suppress the smoke and charred residue for PBa, and acts as an excellent protective layer, limiting the shift of smoke from spreading through the blaze. TOC indicates the total oxygen mass consumed by the polymers during the cone calorimeter test.

Moreover, as shown in Figure 7d, when the TOC of burning composite materials is below that of neat PBa, it indicates that a lower amount of heat is released during combustion, which corresponds to a smaller fire risk for neat PBa. Meanwhile, the decomposition of D-fu can release non-combustible gases and form a stable carbon layer. Furthermore, the non-combustible gases dilute the volatile fuel, and the char layer also isolates the oxygen to protect the internal matrix. In short, these results indicate that the prepared PBa/PD-fu composites improved the flame retardance of PBa.

### 3.5. Flame Retardance Mechanism

#### 3.5.1. Condensed Phase Analysis

The decomposition products of PBa and PBa/PD-fu composites in the condensed phases obtained after CONE tests were determined through SEM and Raman spectra to study possible flame-retardant mechanisms. The charred layers of neat PBa are broken and cracked (Figure 8a). Interestingly, it can be observed from Figure 8b–d that PBa/PD-fu-1%, PBa/PD-fu-3% and PBa/PD-fu-5% possess dense, continuous, and compact char surfaces. This is due to the higher incorporation of D-fu, which promotes the formation of a high-quality char layer during the burning process. Figure 8(a_1_–d_2_) show the morphology of the outside and internal structures of original PBa and PBa/PD-fu charred layers. The charred layer of neat PBa is loose and fragile, exhibiting an open-cell structure, which cannot inhibit the permeation of CO and CO_2_ gases. Hence, PBa is not well-protected, as shown in Figure 8(a_1_). By contrast, Figure 8(b_1_–d_1_) show that the outside structure of the charred layers becomes a more continuous and compact void-free surface after the addition of D-fu. In addition, the inner structure of the charred layers of neat PBa is irregular and possesses several holes. In particular, the alveolate internal structure of the charred layers of PBa/PD-fu-5% becomes more continuous and consistent with tight and compact carbon layers, as shown in Figure 8(d_2_). This implies that D-fu plays a considerable role in the formation of surface morphology and internal structure of the high-quality char, which provides an efficient layer to inhibit the transfer of O_2_, heat and mass among the flaming regions and the unburnt inner areas, contributing to an obvious enhancement in flame-retardant properties.

The decomposition products of PBa and PBa/PD-fu composites in the condensed phases obtained after CONE tests were determined through SEM and Raman spectra to study possible flame-retardant mechanisms. The charred layers of neat PBa are broken and cracked (Figure 8a). Interestingly, it can be observed from Figure 8b–d that PBa/PD-fu-1%, PBa/PD-fu-3% and PBa/PD-fu-5% possess dense, continuous, and compact char surfaces. This is due to the higher incorporation of D-fu, which promotes the formation of a high-quality char layer during the burning process. Figure 8(a_1_–d_2_) show the morphology of the outside and internal structures of original PBa and PBa/PD-fu charred layers. The charred layer of neat PBa is loose and fragile, exhibiting an open-cell structure, which cannot inhibit the permeation of CO and CO_2_ gases. Hence, PBa is not well-protected, as shown in Figure 8(a_1_). By contrast, Figure 8(b_1_–d_1_) show that the outside structure of the charred layers becomes a more continuous and compact void-free surface after the addition of D-fu. In addition, the inner structure of the charred layers of neat PBa is irregular and possesses several holes. In particular, the alveolate internal structure of the charred layers of PBa/PD-fu-5% becomes more continuous and consistent with tight and compact carbon layers, as shown in Figure 8(d_2_). This implies that D-fu plays a considerable role in the formation of surface morphology and internal structure of the high-quality char, which provides an efficient layer to inhibit the transfer of O_2_, heat and mass among the flaming regions and the unburnt inner areas, contributing to an obvious enhancement in flame-retardant properties.

The decomposition products of PBa and PBa/PD-fu composites in the condensed phases obtained after CONE tests were determined through SEM and Raman spectra to study possible flame-retardant mechanisms. The charred layers of neat PBa are broken and cracked (Figure 8a). Interestingly, it can be observed from Figure 8b–d that PBa/PD-fu-1%, PBa/PD-fu-3% and PBa/PD-fu-5% possess dense, continuous, and compact char surfaces. This is due to the higher incorporation of D-fu, which promotes the formation of a high-quality char layer during the burning process. Figure 8(a_1_–d_2_) show the morphology of the outside and internal structures of original PBa and PBa/PD-fu charred layers. The charred layer of neat PBa is loose and fragile, exhibiting an open-cell structure, which cannot inhibit the permeation of CO and CO_2_ gases. Hence, PBa is not well-protected, as shown in Figure 8(a_1_). By contrast, Figure 8(b_1_–d_1_) show that the outside structure of the charred layers becomes a more continuous and compact void-free surface after the addition of D-fu. In addition, the inner structure of the charred layers of neat PBa is irregular and possesses several holes. In particular, the alveolate internal structure of the charred layers of PBa/PD-fu-5% becomes more continuous and consistent with tight and compact carbon layers, as shown in Figure 8d_2_. This implies that D-fu plays a considerable role in the formation of surface morphology and internal structure of the high-quality char, which provides an efficient layer to inhibit the transfer of O_2_, heat and mass among the flaming regions and the unburnt inner areas, contributing to an obvious enhancement in flame-retardant properties.

Moreover, intumescent flame retardants (IFR) can increase the degradation reaction of polymer matrices [44,45,46]. The D-fu in PBa material can form polyphosphoric acid after the initial decomposition stage; this can modify the crosslinking structure of the burned composites, depending on the appearance of phosphazene structure and introduces many phosphorus-containing groups into the burned composites to accelerate high quality char residues. Furthermore, the dehydration process is decided by the -OH group in PBa to produce a cross-linking structure during initial degradation. Meanwhile, continuous and compact char residues are formed to protect the interior structure of the material. Moreover, IFR renders a cooperated fire-retardance performance between the acid, gas, and carbonization source [47]. Here, the acidic source is D-fu, whereas both PBa and D-fu act as the sources for the gas and char. During combustion, the acidic source is helpful to the quality improvement of the char layer of PBa. There seems to be no doubt that continuous compact charred layers are significant in inhibiting heat transfer, resisting the mass loss and delaying the emitting of pyrolysis materials. The non-combustible gases (H_2_O, NH_3_ and CO_2_) are formed and can dilute oxygen concentration, thereby improving the fire-resistance performance. In summary, SEM results demonstrated that the intumescent charred residues can effectively delay the thermal-mass transfer between the gaseous phases and solid phases, supplying a vital fire shield for the lower materials during burning [48].

**Figure 9:**
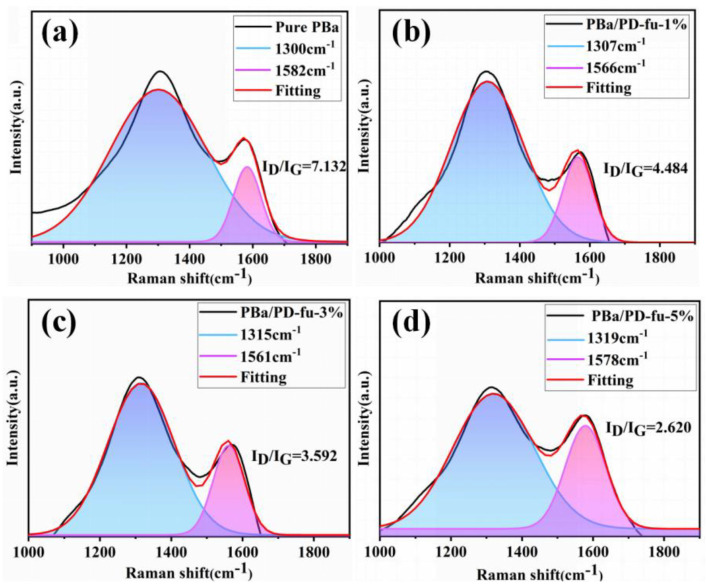
Raman spectra of charred residue of (**a**) PBa, (**b**) PBa/PD-fu-1%, (**c**) PBa/PD-fu-3%, and (**d**) PBa/PD-fu-5% after the CONE test.

Furthermore, Raman spectroscopy was employed to measure the chemical structure of the char residue obtained from CONE tests. The degree of graphitization is the ratio of I_D_/I_G_, reflecting the defect level or integrity of carbonaceous materials [49,50]. In general, a higher graphitization degree of the char layer indicates a greatly restrained performance on O_2_ and mass transfer, when materials decompose. As shown in Figure 9, the char of PBa/PD-fu-5% shows a minimum I_D_/I_G_ value of 2620, corresponding to a high graphitization degree. This reveals that the char residue of PBa/PD-fu is much more structurally integrated than the neat PBa. One should note that these results are consistent with our previous SEM observations.

#### 3.5.2. Gas Phase Analysis

**Figure 10:**
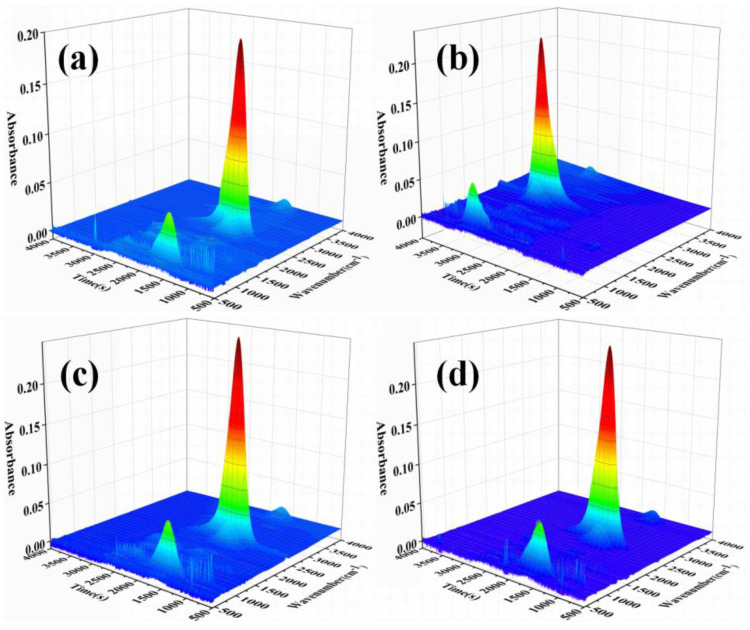
3D TG-FTIR spectra of thermal decomposed compounds of (**a**) PBa, (**b**) PBa/PD-fu-1%, (**c**) PBa/PD-fu-3%, and (**d**) PBa/PD-fu-5%.

**Figure 11:**
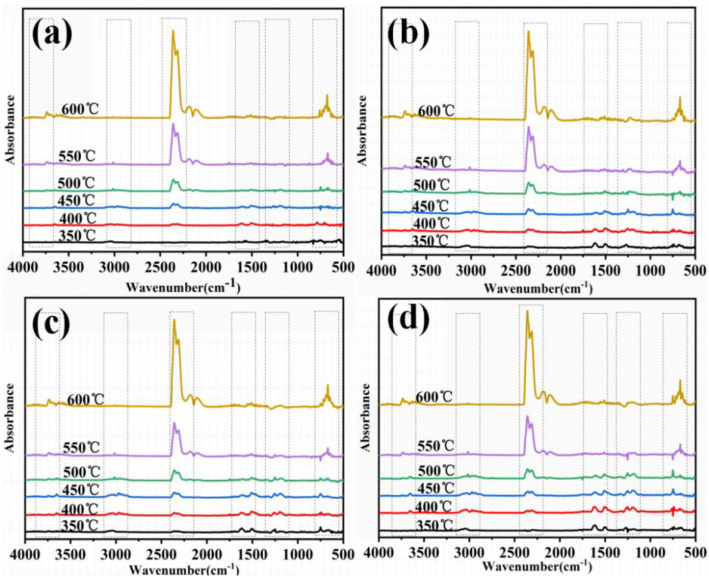
FTIR spectra of (**a**) PBa, (**b**) PBa/PD-fu-1%, (**c**) PBa/PD-fu-3% and (**d**) PBa/PD-fu-5% at various temperatures.

**Figure 12:**
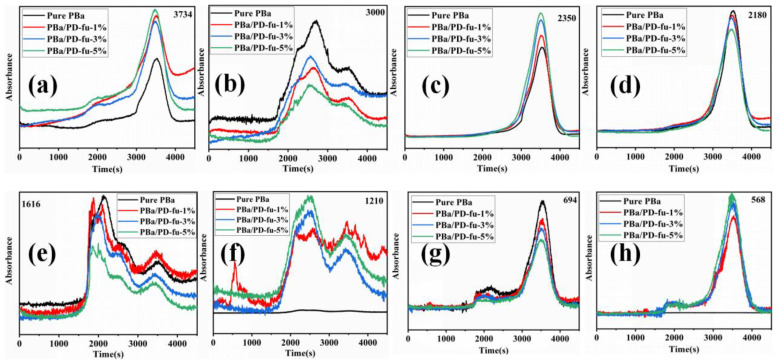
The analysis of pyrolysis products for neat PBa, PBa/PD-fu-1%, PBa/PD-fu-3% and PBa/PD-fu-5%: (**a**) water and ammonia gas, (**b**) alkane-containing compounds, (**c**) CO_2_, (**d**) CO, (**e**) and (**g**) aromatic compounds, (**f**) and (**h**) P–O and P=O compounds.

**Figure 13:**
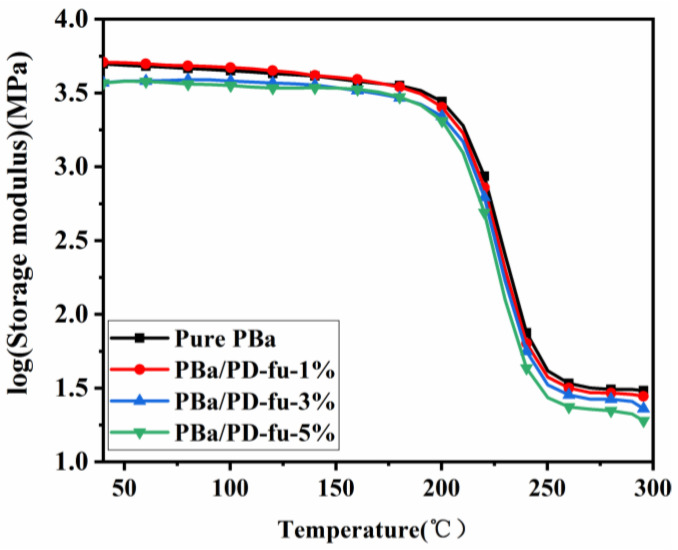
Storage moduli of neat PBa, PBa/PD-fu-1% and PBa/PD-fu-3% and PBa/PD-fu-5%.

To study the role of D-fu in the gaseous phase during burning, the TG-FTIR technique was employed to analyze the pyrolysis products of PBa and PBa/PD-fu composites. The three-dimensional (3D) TG-FTIR spectra were recorded during the thermal decomposition procedure, as shown in Figure 10. We can see that the TG-FTIR spectra of both PBa/PD-fu composites and pure PBa are similar to each other. Specifically, the TG-FTIR spectra of the major pyrolysis products of pure PBa are located at 3700–3800 cm^−1^, 2900–3000 cm^−1^, 2350 cm^−1^ and 1500–1750 cm^−1^, on account of H_2_O and NH_3_-including compounds, alkane-containing compounds, carbon dioxide, and aromatic compounds, respectively [51,52]. Figure 11b–d demonstrate that the P-O and P=O groups in D-fu are thermally degraded at lower temperatures. Meanwhile, it is obvious that the incorporation of D-fu plays a vital role in improving the pyrolysis of the original material. The characterized peak intensity of FTIR spectra with respect to time for the decomposition products is displayed in Figure 12a–h. First, the intensity of the characteristic peaks increases with the addition of D-fu, as shown in Figure 12a; this is caused by the generation of water and ammonia gas. Meanwhile, the intensity of the CO_2_ peak in TG-FTIR spectra of PBa/PD-fu composites is reduced (Figure 12c). These incombustible gases weaken the fuel and O_2_ concentrations in the gaseous phase, consequently suppressing the combustion to a certain degree. In addition, the peaks in Figure 13b,d,e,g correspond to the alkane-containing compounds, CO, and aromatic compounds, respectively [53]. Moreover, PBa/PD-fu polymers produced fewer unstable decomposing compounds than did the neat PBa. These compounds are primarily produced by the chain scission of PBa. This suggests that the loading of D-fu can improve the flame retardance of the PBa matrix. Most importantly, the aforementioned new peaks can be assigned to the stretching vibrations of P-O and P=O bonds, respectively (Figure 12f,h) [54]. These bands imply that certain phosphorous-containing groups in D-fu are degraded into gas components during the thermal decomposition process. In the gas phase, the role of continuous burning of polymeric materials is the generation of H• and HO• radicals. D-fu in the PBa matrix generates PO• radicals during the burning process and can capture HO• and H• radicals in the flame zone [55,56]. In summary, the evidently improved flame-retardant properties of PBa/PD-fu composites can be attributed to the “P-N”-containing flame-retardant system possessing the physical-inhibit effect of charred layers, as well as the dilution effect of non-flammable gases in combustion.

### 3.6. Dynamic Mechanical Analysis

The aforementioned results demonstrate that the loading of D-fu into the PBa matrix improves the flame retardancy of the composite materials. Nevertheless, the thermal mechanical properties of composites usually deteriorate with an improvement in fire resistance, especially for a P–N flame-retardant system. Therefore, the thermal behaviors of the D-fu-loaded PBa matrix, including Tg and the storage modulus, were analyzed by DMA.

**Figure 14:**
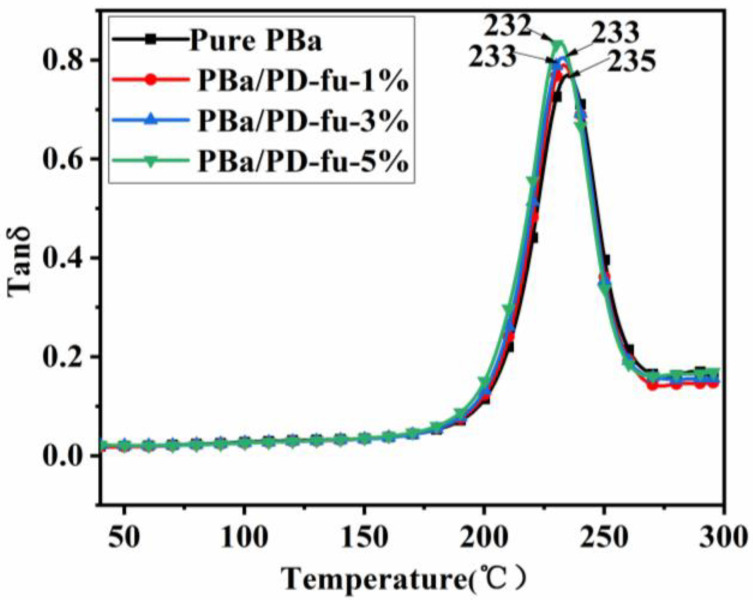
Tan δ vs. temperature curves of neat PBa, PBa/PD-fu-1%, PBa/PD-fu-3% and PBa/PD-fu-5%.

Figure 13 and Figure 14 show the relationship of storage modulus and loss angle tangent (tanδ) with temperature, respectively. Figure 13 demonstrates that the storage moduli of PBa/PD-fu composites are slightly lower than those of neat PBa, which can be attributed to the fact that the inert D-fu lowers the cross-linking density. The peak of tanδ shifts slightly to a lower temperature (Figure 14). In particular, the Tg of neat PBa is 235 °C, whereas the Tg of PBa/PD-fu composite reduces to 233 °C with the incorporation of 1% and 3% D-fu (Figure 14). Meanwhile, the PBa/PD-fu-5% shows a Tg of 232 °C. In short, the addition of D-fu slightly decreases the Tg of the benzoxazine polymer, which is attributed to the fact that the existence of D-fu in PBa composites hinders the ring-opening cross-linking degree of Ba monomers because of steric-hindrance effects, resulting in a reduction in the crosslink density of PBa [49,50]. However, the Tg of PBa/PD-fu composites is still higher than 200 °C, which is higher than most conventional thermosetting resins. Hence, D-fu does not render any significant influence on the working temperature and application environment of PBa.

## 4. Conclusions

In summary, a new type of phosphorous-containing bio-based furfurylamine type benzoxazine, named D-fu, was prepared and characterized using FTIR and ^1^H-NMR. Our results show that the incorporation of D-fu slightly decreases the initial curing temperature of the Ba/D-fu mixture and enhances the char-formation performance of PBa/PD-fu composites, as well as reducing the HRR, TOC, and content of toxic gases during combustion. The LOI value and UL-94 level of PBa composite materials have been significantly improved with the addition of D-fu. The analysis of fire retardance mechanism revealed that the thermal degradation of PBa catalyzed by D-fu can improve the quality and stability of the charred layers in the condensed phase and can accelerate the degradation in the gaseous phase to produce non-flammable gases. Finally, the results of DMA confirmed the prepared PBa composites still possess a sufficiently high Tg. Thus, the synthesized D-fu can enlarge the application of PBa in the fields of fire safety and high-performance materials.

## Figures and Tables

**Table 1:** The curing parameters for Ba and Ba/D-fu systems at a heating rate of 10 °C/min.

Sample	Ti (°C)	Tp (°C)	Tf (°C)
Ba	210	234	286
Ba/D-fu-1%	206	230	285
Ba/D-fu-3%	206	232	285
Ba/D-fu-5%	204	229	281

**Table 2:** The TGA results for PBa and composites in air and N_2_.

Sample	T_onset_ (°C)		T_max_ (°C)		Char Residue (%)			
					700 °C		800 °C	
	Air	N_2_	Air	N_2_	Air	N_2_	Air	N_2_
PBa	376	355	408	410	5 ± 0.13	36 ± 0.33	0.34 ± 0.17	34 ± 0.16
PBa/PD-fu-1%	362	353	389	400	19 ± 0.25	37 ± 1.30	1.26 ± 0.14	36 ± 0.91
PBa/PD-fu-3%	366	348	382	388	14 ± 0.32	37 ± 0.49	2.34 ± 0.11	36 ± 0.41
PBa/PD-fu-5%	366	351	378	380	12 ± 0.50	38 ± 0.60	2.64 ± 0.26	37 ± 0.98

**Table 3:** CONE parameters and LOI results of PBa and PBa/PD-fu polymers.

Sample	TTI (s)	PHRR (kW/m^2^)	Mass Loss (%)	FPI (s·m^2^/kW)	LOI (%)	UL-94
Pure PBa	83	330	77.9%	0.25	21	NR
PBa/PD-fu-1%	82	332	74.4%	0.25	25	V-1
PBa/PD-fu-3%	76	258	73.7%	0.29	29	V-0
PBa/PD-fu-5%	75	258	71.7%	0.29	34	V-0

## Data Availability

Not applicable.
